# CD80 Expression on Tumor Cells Alters Tumor Microenvironment and Efficacy of Cancer Immunotherapy by CTLA-4 Blockade

**DOI:** 10.3390/cancers13081935

**Published:** 2021-04-16

**Authors:** Julie Vackova, Ingrid Polakova, Shweta Dilip Johari, Michal Smahel

**Affiliations:** 1 Department of Genetics and Microbiology, Faculty of Science, Charles University, BIOCEV, 252 50 Vestec, Czech Republic; julie.vackova@natur.cuni.cz (J.V.); ingrid.polakova@natur.cuni.cz (I.P.); joharis@natur.cuni.cz (S.D.J.); 2 Department of Cell Biology, Faculty of Science, Charles University, BIOCEV, 252 50 Vestec, Czech Republic

**Keywords:** CD80, CTLA-4, PD-L1, tumor-infiltrating lymphocytes, cancer, immune checkpoint blockade

## Abstract

**Simple Summary:**

The recent discovery of immune checkpoint inhibitors constituted a breakthrough in cancer treatment, but most patients are resistant to this therapy. Although the co-stimulatory molecule cluster of differentiation (CD) 80 has been detected in several types of tumor cells, its role in the tumor microenvironment and its sensitivity to immune checkpoint blockade are unclear. We, therefore, introduced a clinically relevant mouse tumor model with deactivated CD80. The deactivation promoted a “hot” tumor microenvironment and enhanced the sensitivity to immune checkpoint blockade with antibody against the cytotoxic T-lymphocyte antigen 4 (CTLA-4). This study contributed to the research into predictive markers to select patients who are suitable for immune checkpoint blockade therapy and suggested the development of a novel cancer immunotherapy based on a tumor-cell-targeted CD80 blockade.

**Abstract:**

Cluster of differentiation (CD) 80 is mainly expressed in immune cells but can also be found in several types of cancer cells. This molecule may either activate or inhibit immune reactions. Here, we determined the immunosuppressive role of CD80 in the tumor microenvironment by CRISPR/Cas9-mediated deactivation of the corresponding gene in the mouse oncogenic TC-1 cell line. The tumor cells with deactivated CD80 (TC-1/dCD80-1) were more immunogenic than parental cells and induced tumors that gained sensitivity to cytotoxic T-lymphocyte antigen 4 (CTLA-4) blockade, as compared with the TC-1 cells. In vivo depletion experiments showed that the deactivation of CD80 switched the pro-tumorigenic effect of macrophages observed in TC-1-induced tumors into an anti-tumorigenic effect in TC-1/dCD80-1 tumors and induced the pro-tumorigenic activity of CD4^+^ cells. Moreover, the frequency of lymphoid and myeloid cells and the CTLA-4 expression by T helper (Th)17 cells were increased in TC-1/dCD80-1- compared with that in the TC-1-induced tumors. CTLA-4 blockade downregulated the frequencies of most immune cell types and upregulated the frequency of M2 macrophages in the TC-1 tumors, while it increased the frequency of lymphoid cells in TC-1/dCD80-1-induced tumors. Furthermore, the anti-CTLA-4 therapy enhanced the frequency of CD8^+^ T cells as well as CD4^+^ T cells, especially for a Th1 subset. Regulatory T cells (Treg) formed the most abundant CD4^+^ T cell subset in untreated tumors. The anti-CTLA-4 treatment downregulated the frequency of Treg cells with limited immunosuppressive potential in the TC-1 tumors, whereas it enriched this type of Treg cells and decreased the Treg cells with high immunosuppressive potential in TC-1/dCD80-1-induced tumors. The immunosuppressive role of tumor-cell-expressed CD80 should be considered in research into biomarkers for the prediction of cancer patients’ sensitivity to immune checkpoint inhibitors and for the development of a tumor-cell-specific CD80 blockade.

## 1. Introduction

The costimulatory molecule cluster of differentiation (CD) 80, which can be expressed on antigen-presenting cells (APCs) or tumor cells, interacts with both costimulatory (CD28) and coinhibitory (cytotoxic T-lymphocyte antigen 4 (CTLA-4)) receptors and regulates the immune response [1,2]. The binding of CD28 and CTLA-4 to CD80 is competitive and regulated by several factors, such as affinity to CD80 and kinetics of CD28 and CTLA-4 expression in T cells [2]. CTLA-4 has approximately ten times higher affinity to CD80 than CD28. However, CTLA-4 is mainly expressed on activated T cells, while CD28 is expressed on T cells constitutively [3]. 

It has been previously reported that the expression level of CD80 may regulate the pro-/anti-oncogenic role of CD80 on tumor cells [4,5,6]. Low levels of CD80 expression serve as a mechanism of tumor escape from immune surveillance due to a higher affinity and, therefore, preferential binding of CTLA-4 to CD80 compared with that for CD28. On the contrary, overexpression of CD80 promotes T cell activation and tumor rejection, and a CD80 deficiency also increases the immunogenicity of tumor cells.

Furthermore, it has been shown that a soluble form of CD80 binds to programmed cell death ligand 1 (PD-L1) and inhibits the PD-1/PD-L1 axis [7]. Moreover, PD-L1 and CD80 interaction in cis, but not in trans, inhibits the immunosuppressive PD-1/PD-L1 and CTLA-4/CD80 axes [8,9]. PD-L1-blocking antibodies prevent CD80/PD-L1 interaction on tumor-associated dendritic cells (DCs) and promote the CD80-mediated anti-tumor immune response [10]. However, PD-L1 blockade may enhance CTLA-4/CD80-mediated immunosuppression in certain settings [9]. 

According to the dataset of The Cancer Genome Atlas (TCGA) PanCancer Atlas Studies, available at the cBioPortal (https://bit.ly/3hE21OQ, accesse on 5 March 2021; 10,953 cancer patients from 32 studies), the *CD80* gene is altered in 2% of patients, including amplifications, deletions, and mutations that may result in unfunctional CD80. A deficiency of CD28/CD80- or CD28/CD86-mediated co-stimulation induces T cell anergy or the establishment of a regulatory T cell (Treg) phenotype [11,12]. Moreover, anergic T cells can serve as precursors to the establishment of peripheral Treg cells [13,14]. Treg cells inhibit the anti-tumor immune response, enhance cancer progression, and mediate resistance to cancer therapy by several mechanisms. For instance, Treg cells secrete the immunosuppressive cytokines and cytotoxic molecules, perforin and granzymes, which target effector immune cells [15,16] and express CD39 and CD73 ectonucleotidases, which produce immunosuppressive adenosine from extracellular adenosine triphosphate (ATP), which is highly abundant in the tumor microenvironment [17,18]. In addition, some inhibitory receptors on Treg cells directly inhibit immune reactions. Surface expression of CTLA-4 is considered a key immunosuppressive mechanism of Treg cells [19]. CTLA-4 inhibits the immune response via a blockade of antigen presentation due to its higher affinity to CD80 in comparison with CD28. Treg cells also decrease the amount of CD80 on the surface of APCs by CTLA-4-mediated trans-endocytosis [20,21]. CTLA-4^−^ Treg cells had impaired immunosuppressive functions and were ineffective in the control of immune responses [22]. The direct role of the lymphocyte-activation gene 3 (Lag3) receptor in Treg immunosuppression has been reported, but this issue is still debatable [23,24]. 

Treg-mediated immunosuppression is tightly regulated. Inducible T cell costimulator (ICOS), glucocorticoid-induced tumor necrosis factor receptor (GITR), or neuropilin 1 (Nrp-1) induce the proliferation and effector functions of Treg cells and their tumor infiltration [25,26]. On the contrary, PD-1 signaling inhibits Treg cell effector functions. Therefore, Treg cells are activated by the blockade of the PD-1/PD-L1 axis, which may induce the hyper-progression of cancer [27,28]. 

Immune checkpoint inhibitors targeting the CTLA-4/CD80 or PD-1/PD-L1 axes constituted a major breakthrough in the treatment of several types of tumors, such as inoperable or metastatic melanoma and non-small-cell lung cancer (NSCLC) [29,30,31,32]. However, many patients are resistant to immune checkpoint blockade. Predictive biomarkers are, thus, necessary to distinguish patients who will benefit from this therapy [33]. 

Regarding the various interacting partners of CD80, with their broad range of effects on anti-tumor immune responses, the role of CD80 in the tumor microenvironment and its impact on the efficacy of cancer therapy need to be elucidated. In the present study, we developed an experimental model of mouse tumors with functional or deactivated CD80 and compared their immunogenicity and sensitivity to immunotherapy. Tumors with CD80 deactivation were more immunogenic and prone to CTLA-4 blockade. 

## 2. Results

### 2.1. CD80 Deactivation Reduced Tumor Growth

**Figure 1:**
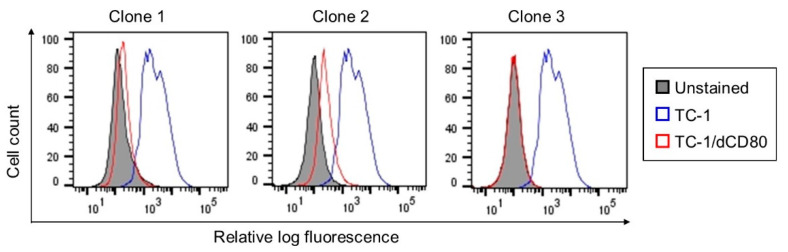
Flow cytometry analysis of co-stimulatory molecule cluster of differentiation (CD) 80 surface expression on the TC-1 cell line and TC-1/dCD80 clones. Unstained cells were used as the negative control.

**Figure 2:**
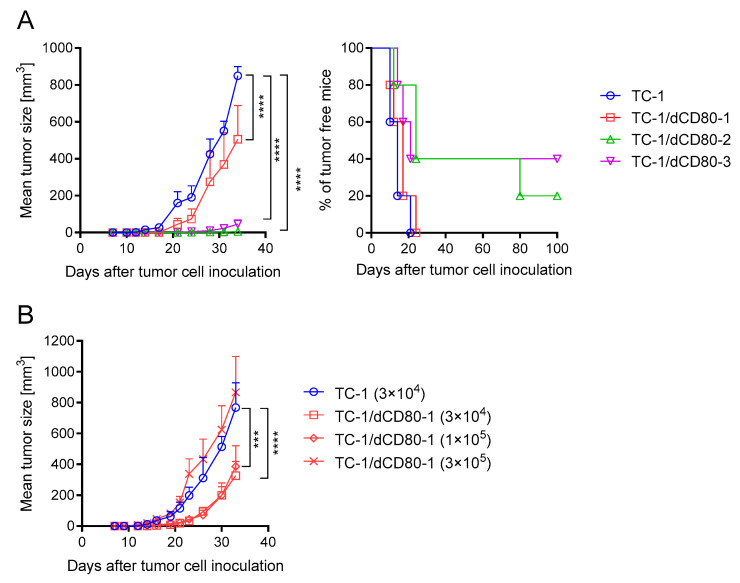
TC-1/dCD80 immunogenicity in comparison with TC-1 cell line. C57BL/6 mice (*n* = 5) were subcutaneously (s.c.) injected with 3 × 10^4^ cells of three TC-1/dCD80 clones (**A**) or three different doses of the TC-1/dCD80-1 clone (**B**). In total, 3 × 10^4^ TC-1 cells were inoculated for comparison. Tumor growth and tumor formation were evaluated. Statistical analysis indicates a comparison with TC-1 cells. Bars indicate ±standard error of mean (SEM); *** *p* < 0.001, **** *p* < 0.0001.

As CD80 expression on tumor cells can influence their immunogenicity, we sought to determine whether CD80 expression on TC-1 cells [34] affects tumor growth, the tumor microenvironment, and the sensitivity to immune checkpoint blockade. Therefore, we generated TC-1 cells with a deactivated CD80 molecule (TC-1/dCD80) using the CRISPR/Cas9 system. As the clones obtained after CD80 deactivation were heterogeneous in their CD80 expression, we selected three clones with apparently reduced CD80 surface expression compared with TC-1 cells (Figure 1) for an analysis of immunogenicity (Figure 2A). Deactivation of CD80 in the TC-1 cell line significantly reduced the growth of tumors induced with all three clones. While TC-1/dCD80 clone 1 (TC-1/dCD80-1) formed tumors in all inoculated mice, clones TC-1/dCD80-2 and -3 generated tumors in 80% and 60% of mice, respectively, and their growth was highly delayed. We, therefore, used the TC-1/dCD80-1 clone in our further study of the tumor microenvironment and immunotherapy response.

We first tested various doses of TC-1/dCD80-1 cells to find a dose inducing tumors of a size that was comparable to the tumors induced with 3 × 10^4^ TC-1 cells. While the growth of the TC-1/dCD80-1-induced tumors after inoculation of 3 × 10^4^ and 1 × 10^5^ cells was slower than that of TC-1-induced tumors, the 3 × 10^5^ dose significantly enhanced the growth of TC-1/dCD80-1-induced tumors and provided tumors of a similar size to those induced with 3 × 10^4^ TC-1 cells (Figure 2B). 

### 2.2. CD80 Deactivation Altered Immune Reactions and Sensitivity to CTLA-4 Blockade 

**Figure 3:**
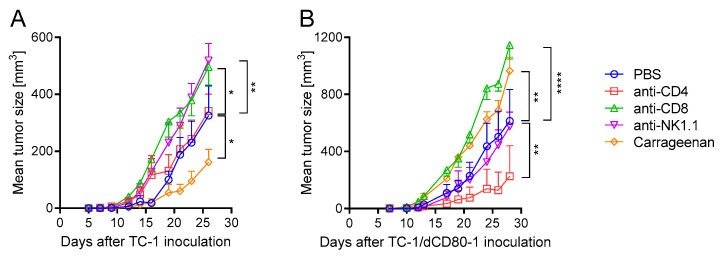
The role of immune cells in tumor growth. CD4^+^, CD8^+^, or natural killer (NK) 1.1^+^ cells were depleted by monoclonal antibodies and macrophages by carrageenan in mice bearing TC-1-induced tumors (**A**) or TC-1/dCD80-1-induced tumors (**B**). Statistical analysis indicates a comparison with the phosphate-buffered saline (PBS)-treated group. Bars indicate ±SEM; * *p* < 0.05, ** *p* < 0.01, **** *p* < 0.0001.

To investigate the impact of the CD80 deactivation in tumor cells on anti-tumor immune reactions, we depleted CD4^+^, CD8^+^, natural killer (NK) 1.1^+^ cells, or macrophages in mice bearing TC-1- and TC-1/dCD80-1-induced tumors (Figure 3). The CD80 deactivation switched the pro-tumor role of macrophages observed in TC-1-induced tumors (Figure 3A) to anti-tumor abilities in TC-1/dCD80-1-induced tumors (Figure 3B). Moreover, the depletion of CD4^+^ cells did not markedly affect the growth of TC-1-induced tumors (Figure 3A) but did reduce the growth of TC-1/dCD80-1-induced tumors (Figure 3B). CD8^+^ cells had an anti-tumor effect regardless of CD80 expression on tumor cells. Depletion of NK1.1^+^ cells significantly enhanced the growth of TC-1-induced tumors, whereas it did not have a significant impact on the growth of TC-1/dCD80-1-induced tumors. 

**Figure 4:**
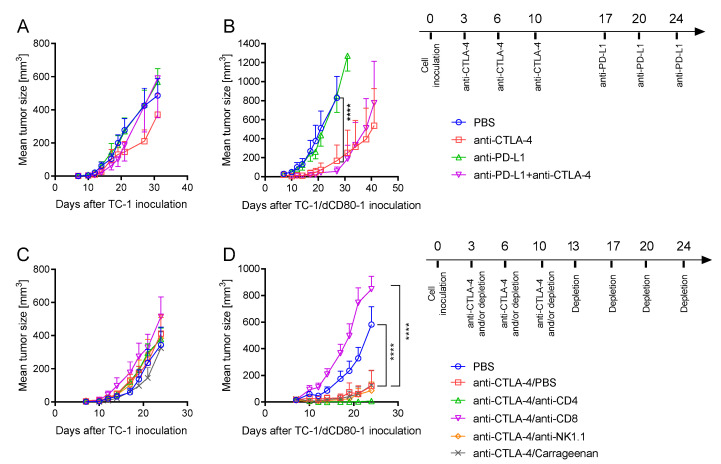
Immunotherapy by immune checkpoint blockade. Mice (*n* = 5) bearing either TC-1- (**A**) or TC-1/dCD80-1-induced tumors (**B**) were treated with anti-cytotoxic T-lymphocyte antigen 4 (CTLA-4), anti-programmed cell death ligand 1 (PD-L1), or a combination of both antibodies. The cells involved in the anti-tumor effect of CTLA-4 blockade were analyzed by depletions of CD4^+^, CD8^+^, NK1.1^+^ cells, or macrophages in mice bearing TC-1-induced tumors (**C**) or TC-1/dCD80-1-induced tumors (**D**). Phosphate-buffered saline (PBS) was used as the control. Statistical analysis indicates a comparison with the anti-CTLA-4/PBS group. Bars indicate ±SEM; **** *p* < 0.0001.

Furthermore, we tested the efficacy of immune checkpoint blockade with anti-CTLA-4 and anti-PD-L1 antibodies in mice bearing TC-1- and TC-1/dCD80-1-induced tumors (Figure 4). Unlike the TC-1-induced tumor growth, which was negligibly reduced by anti-CTLA-4 (Figure 4A), TC-1/dCD80-1-induced tumor growth was markedly inhibited by the antibody (Figure 4B). The anti-PD-L1 antibody had no impact on either TC-1- (Figure 4A) or TC-1/dCD80-1-induced tumor growth (Figure 4B). Next, we hypothesized that the CTLA-4/CD80 axis may inhibit the effect of PD-1/PD-L1 blockade. Therefore, we simultaneously treated mice with PD-L1 and CTLA-4 blockades. However, these combined blockades did not outperform the anti-tumor effect of CTLA-4 blockade, suggesting that CTLA-4-mediated immunosuppression does not cause resistance to PD-L1 blockade.

To determine the immune cells involved in the therapeutic outcome of CTLA-4 blockade, we depleted CD4^+^, CD8^+^, NK1.1^+^ cells, or macrophages in mice bearing TC-1- and TC-1/dCD80-1-induced tumors (Figure 4C,D). None of the depletions of immune cell subpopulations significantly influenced the growth of TC-1-induced tumors in mice treated with CTLA-4 blockade (Figure 4C). The depletion of CD8^+^ cells abrogated the therapeutic effect of CTLA-4 blockade in TC-1/dCD80-1-induced tumors (Figure 4D). On the contrary, anti-CD4 treatment synergized with CTLA-4 blockade to reduce the growth of these tumors. The depletion of NK1.1^+^ cells or macrophages did not influence the efficacy of CTLA-4 blockade against TC-1/dCD80-1 tumors.

### 2.3. CD80 Deactivation and CTLA-4 Blockade Altered Tumor Microenvironment

**Figure 5:**
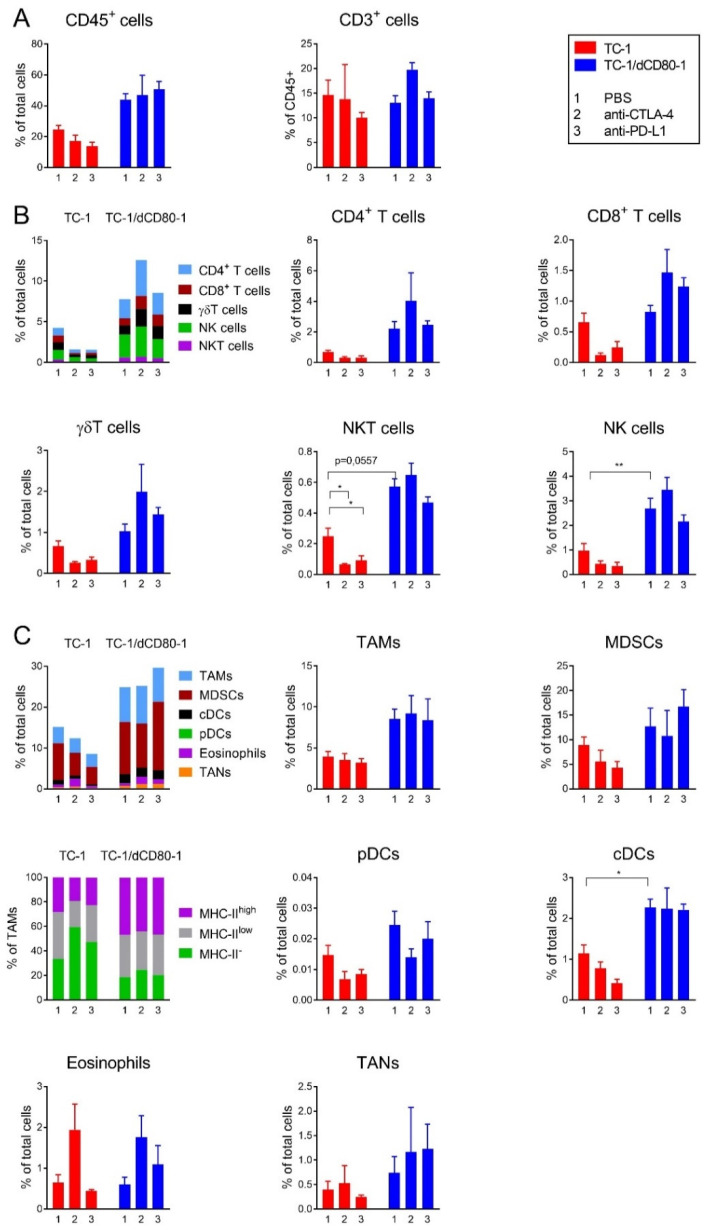
Flow cytometry analysis of immune cells infiltrating TC-1- and TC-1/dCD80-1-induced tumors. Mice (*n* = 4) were treated with anti-CTLA-4 or anti-PD-L1 and PBS was used as the control. The total infiltration by immune cells and the proportion of lymphocytes were determined using CD45 and CD3 markers, respectively (**A**). Cell subpopulations were stained with panels of antibodies detecting either lymphoid (**B**) or myeloid cells (**C**). Bars indicate ±SEM; * *p* < 0.05, ** *p* < 0.01.

We next analyzed the microenvironment of TC-1- and TC-1/dCD80-1-induced tumors by flow cytometry to further characterize the immune cells that contributed to the reduced growth of tumors with deactivated CD80 and mediated the effect of immune checkpoint blockade with anti-CTLA-4 or anti-PD-L1 (Figure 5). Deactivation of CD80 in tumor cells approximately doubled the infiltration of CD45^+^ cells, but the proportion of T lymphocytes was not substantially altered (Figure 5A). The frequency of most subpopulations of immune cells in tumors was affected by CD80 deactivation. In lymphoid cells (Figure 5B), the frequency of natural killer (NK) cells (CD45^+^, CD3^−^, γδ T cell receptor (TCR)^−^, NK1.1^+^) and natural killer T (NKT) cells (CD45^+^, CD3^+^, γδTCR^−^, NK1.1^+^) was markedly enhanced, and the frequency of CD4^+^ T cells (CD45^+^, CD3^+^, γδTCR^−^, NK1.1^−^, CD4^+^) was also increased in TC-1/dCD80-1- compared with that in TC-1-induced tumors. Similarly, the frequency of most myeloid cells was elevated in TC-1/dCD80-1- compared with that in TC-1-induced tumors (Figure 5C), such as tumor-associated macrophages (TAMs; CD45^+^, CD11b^+^, Ly6G^−^, F4/80^+^, SSC^lo^), classical dendritic cells (cDCs; CD45^+^, CD11c^+^, F4/80^−^, Ly6C^−^, CD317^−^), plasmacytoid dendritic cells (pDCs; CD45^+^, CD11c^+^, CD11b^−^, F4/80^−^, Ly6C^+^, CD317^+^), myeloid-derived suppressor cells (MDSCs; CD45^+^, CD11b^+^, Ly6G^−^, F4/80^−^, Ly6C^hi^), and tumor-associated neutrophils (TANs; CD45^+^, CD11b^+^, CD11c^−^, Ly6C^int^, Ly6G^+^).

Furthermore, CD80 deactivation affected the proportions of M1 and M2 macrophages within the TAM subpopulation, as assessed by major histocompatibility complex (MHC)-II expression (Figure 5C). The frequency of MHC-II^hi^ M1 macrophages was markedly increased, whereas the frequency of MHC-II^−^ M2 macrophages was slightly downregulated in TC-1/dCD80-1- compared with that in TC-1-induced tumors. 

CTLA-4 and PD-L1 blockades considerably decreased the infiltration of most immune cells into TC-1-induced tumors, but this effect was significant only for NKT cells (Figure 5B). On the contrary, CTLA-4 blockade substantially increased the infiltration of lymphoid cells into TC-1/dCD80-1-induced tumors, such as CD4^+^ or CD8^+^ T cells, γδT cells, and NK cells, and simultaneously decreased the infiltration of pDCs. Moreover, CTLA-4 blockade enhanced the infiltration of eosinophils (CD45^+^, CD11b^+^, CD11c^−^, Ly6G^−^, SSC^hi^) into tumors, regardless of CD80 expression on tumor cells, and increased the frequency of MHC-II^−^ M2 macrophages within the subpopulation of TAMs in TC-1-induced tumors.

PD-1 expression, a marker of immune cell activation or exhaustion, was significantly altered in some subpopulations of leucocytes after immunotherapy (Figure S1). CTLA-4 blockade enhanced PD-1 expression on γδT cells, cDCs, and eosinophils, and PD-L1 blockade achieved the same on CD4^+^ T cells in TC-1-induced tumors. Furthermore, CTLA-4 blockade significantly promoted PD-1 expression on TAMs in TC-1- as well as TC-1/dCD80-1-induced tumors. In addition, this treatment slightly downregulated PD-1 on CD8^+^ T cells in TC-1-induced tumors, whereas it had the opposite effect on TC-1/dCD80-1-induced tumors. Next, CTLA-4 blockade downregulated PD-1 on TANs, which was significant in TC-1/dCD80-1-induced tumors. PD-L1 blockade did not have a significant impact on PD-1 expression on myeloid cells.

Taken together, CD80 deactivation increased numbers of both lymphoid and myeloid cells infiltrating tumors and was associated with further enhancement of lymphoid cells after treatment with the CTLA-4 antibody.

### 2.4. CD80 Deactivation Resulted in Increased CTL4-4 Expression on Th17 T Cells and Enhanced Frequency of Th1 Cells in Tumors after CTLA-4 Blockade

**Figure 6:**
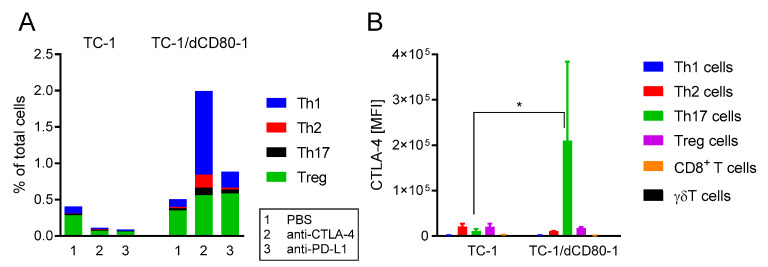
Flow cytometry analysis of the CD4^+^ T cell subpopulations in TC-1- and TC-1/dCD80-1-induced tumors. Tumors (*n* = 4) were treated with the anti-CTLA-4 or anti-PD-L1 antibodies and PBS was used as the control. The frequency of CD4^+^ T cell subsets (**A**) and surface expression of CTLA-4 on T cells in tumors (**B**) was evaluated. Bars indicate ± SEM; MFI—median fluorescent intensity; * *p* < 0.05.

As the above-mentioned depletion of CD4^+^ cells synergized with immunotherapy, we analyzed subsets of tumor-infiltrating CD4^+^ T cells (CD45^+^, CD3^+^, γδTCR^−^, CD4^+^; Figure 6). Treg cells (CD25^+^, Foxp3^+^) were the most abundant CD4^+^ T cells, while the frequency of T helper (Th)1 (IFN-γ^+^), Th2 (IL-4^+^), and Th17 (IL-17A^+^) cells was relatively low in untreated tumors (Figure 6A). Therapy with anti-CTLA-4 or anti-PD-L1 downregulated all CD4^+^ T cell subsets in TC-1-induced tumors. On the contrary, immunotherapy increased the frequency of CD4^+^ T cell subsets in TC-1/dCD80-1-induced tumors, particularly CTLA-4 blockade, which markedly enhanced the frequency of Th1 cells, while the frequency of Th2, Th17, and Treg cells was slightly upregulated in these tumors. These findings indicate that the anti-tumor effect of CTLA-4 blockade in TC-1/dCD80-1-induced tumors was associated with a substantially increased frequency of Th1 cells in these tumors.

In order to assess which T cell subsets were the main target of CTLA-4 blockade in the tumor microenvironment, we next evaluated the level of CTLA-4 expression on T cells. In non-treated tumors, CTLA-4 was mainly expressed on Th2 cells, Th17 cells, and Treg cells, whereas its expression was negligible on Th1, γδT, and CD8^+^ T cells (CD45^+^, CD3^+^, γδTCR^−^, CD8^+^; Figure 6B). In TC-1/dCD80-1-induced tumors, CTLA-4 expression was significantly upregulated on Th17 cells, which might be associated with the enhanced sensitivity of these tumors to CTLA-4 blockade. 

### 2.5. CD80 Deactivation and CTLA-4 Blockade Affected Heterogeneity of Tumor-Infiltrating Treg Cells

**Figure 7:**
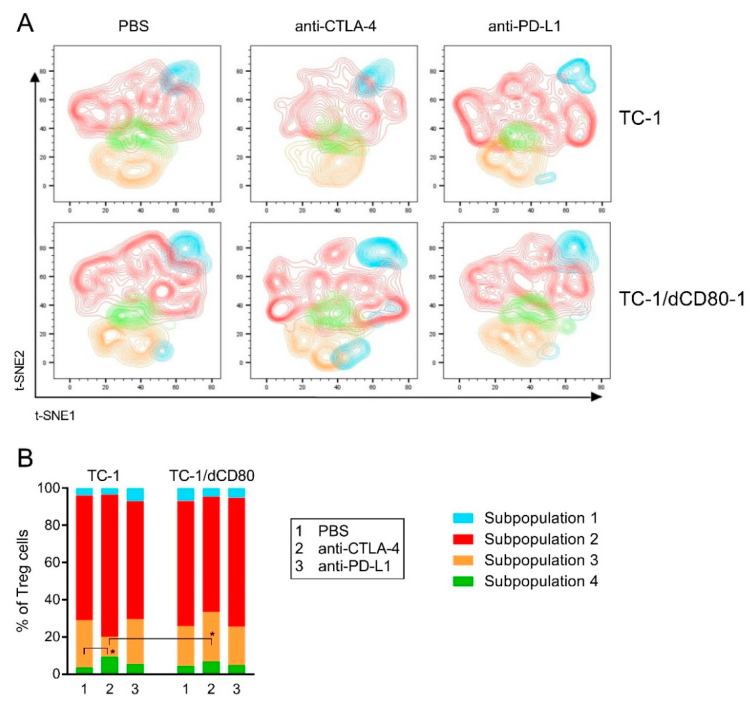
Diversity of Treg cells. In cells isolated from tumors (*n* = 4) treated with PBS, anti-CTLA-4, or anti-PD-L1, distinct Treg subpopulations were identified by the FlowSOM algorithm in the concatenate of all groups of samples and projected into t-distributed stochastic neighbor embedding (t-SNE) plots (**A**). The frequency of Treg subpopulations was statistically evaluated (**B**). * *p* < 0.05.

As Treg cells were the most abundant CD4^+^ subpopulation and their activity may critically influence the effect of immunotherapy, we analyzed the heterogeneity of Treg cells with the unsupervised clustering algorithm FlowSOM using markers of Treg cell activation and effector functions (CTLA-4, GITR, ICOS, Lag3, CD73, granzyme B (GrzB), and Nrp-1; Figure 7). We automatically generated four clusters that represent subsets of Treg cells with a distinct immunosuppressive capacity based on the intensity of expression of the respective markers—subpopulation 1 (CTLA-4^hi^, GITR^hi^, ICOS^hi^, Lag3^lo^, CD73^−^, GrzB^+^, Nrp-1^lo^) and subpopulation 2 (CTLA-4^hi^, GITR^hi^, ICOS^hi^, Lag3^lo^, CD73^+^, GrzB^+^, Nrp-1^lo^) with high immunosuppressive potential, and subpopulation 3 (CTLA-4^−^, GITR^hi^, ICOS^hi^, Lag3^+^, CD73^+^, GrzB^+^, Nrp-1^lo^) and subpopulation 4 (CTLA-4^lo^, GITR^lo^, ICOS^lo^, Lag3^−^, CD73^+^, GrzB^+^, Nrp-1^+^) with weak immunosuppressive potential—and projected the distribution of these subpopulations into t-distributed stochastic neighbor embedding (t-SNE) plots (Figure 7A). Interestingly, CD80 deactivation partially changed the distribution of Treg subpopulation 1 in the t-SNE plot, and this effect was enhanced by anti-CTLA-4 treatment. Subpopulation 2 was the most abundant phenotype within Treg cells in tumors, followed by subpopulation 3 (Figure 7B). CTLA-4 blockade significantly downregulated the frequency of Treg cells with subpopulation 3 and markedly upregulated subpopulations 2 and 4 in TC-1-induced tumors. The proportion of Treg subpopulation 3 was increased in TC-1/dCD80-1-induced tumors treated with CTLA-4 blockade, which resulted in a significant difference in this subpopulation in comparison with the TC-1-induced tumors treated with the same antibody. These data show that CD80 deactivation in tumor cells reduced the immunosuppressive potential of Treg cells after tumor treatment with CTLA-4 blockade. 

Next, we evaluated the effect of CD80 deactivation in tumor cells and immune checkpoint blockade on the expression of Treg markers by the automatically generated Treg subpopulations (Figure S2). Although CD80 deactivation did not have a significant impact on the expression of most markers expressed by the Treg clusters, CD73 expression was significantly downregulated in subpopulation 2 in TC-1/dCD80-1-induced tumors compared with the same subpopulation in the TC-1-induced tumors. Immunotherapy significantly affected the expression of several markers in the Treg subsets, such as the expression of CTLA-4, GITR, CD73, and GrzB in TC-1-induced tumors and the expression of GITR in the TC-1/dCD80-1-induced tumors. These data suggest that CD80 expression in tumor cells and immune checkpoint blockade might have altered immunosuppressive potential within distinct Treg cell subsets.

## 3. Discussion

Immune checkpoint blockade is a promising approach to cancer treatment despite its lack of efficacy in most patients. Markers for the prediction of immune checkpoint blockade efficacy are, therefore, being intensively investigated [35]. CD80 is expressed in various human tumors, such as melanoma, colon adenoma, and gastric adenocarcinoma [4,36,37], as well as in oncogenic cell lines, for instance, cell lines derived from melanoma, colorectal carcinoma, Burkitt’s lymphoma, and gastrointestinal cancer [6,38]. CD80 expression has also been reported in the mouse oncogenic TC-1 cell line used in this study [34]. As CD80 may either stimulate or inhibit the immune response [1,2], we tested the role of CD80 expression on TC-1 cells in the oncogenicity and efficacy of CTLA-4 and/or PD-L1 blockades. Previously, it has been shown that CD80 silencing, as well as overexpression, in tumor cells could inhibit their oncogenicity [6]. In line with that report, CD80 deactivation in TC-1 cells reduced tumor formation and growth in our study. However, the role of CD80 may differ in various tumor types. In contrast to the aforementioned study, deactivation of CD80 expression or its neutralization by an anti-CD80 antibody has promoted the expansion of colonic neoplasia in mice and reduced T cell cytotoxicity [37]. 

Here, we showed that CD80 deactivation in tumor cells affected the pro-/anti-tumorigenic role of distinct immune cell populations. For instance, NK1.1^+^ cells lost the anti-tumorigenic function in mice bearing TC-1/dCD80-1- compared with those with TC-1-induced tumors. Our data correspond to the observation of an NK cell-mediated reduction of CD80^high^ tumor growth [5]. Surprisingly, we observed that CD80 deactivation in tumor cells markedly enhanced infiltration by NK and NKT cells, although the depletion of NK1.1^+^ cells did not affect TC-1/dCD80-1-induced tumor growth. Next, macrophages gained an anti-tumorigenic function after CD80 deactivation in TC-1 cells, which is in agreement with the enhanced frequency of M1 macrophages in TC-1/dCD80-1-induced tumors. In line with this result, the blockade of the CTLA-4/CD80 axis with ipilimumab in melanoma patients has induced a switch in macrophage polarization from the M2 to the M1 phenotype [39]. As CD4^+^ T cells and activated NKT cells have been reported to promote macrophage polarization into the M1 phenotype [40,41,42], enhanced infiltration of these cells might alter macrophage polarization in TC-1/dCD80-1-induced tumors. Furthermore, CD4^+^ cells became pro-tumorigenic in TC-1/dCD80-1-induced tumors, while CD8^+^ cells controlled tumor growth regardless of CD80 expression. It has been reported that CD4^+^ T cells preferentially express CTLA-4 and, therefore, have higher immunosuppressive potential compared with that of CD8^+^ T cells [43]. Moreover, CD80 deactivation in tumor cells promoted tumor infiltration with APCs in our model. CD28 signaling induced by APC-mediated co-stimulation has been shown to enhance CTLA-4 expression, predominantly on the Th17 cell subset [44]. In our study, the APCs in the TC-1/dCD80-1-induced tumors might enhance T cell activation and CTLA-4 expression on Th17 cells.

CTLA-4 blockade did not significantly reduce the growth of TC-1-induced tumors, while it did markedly inhibit the growth of TC-1/dCD80-1-induced tumors. Furthermore, PD-L1 blockade was ineffective as a single therapy, and it did not enhance the effect of CTLA-4 blockade in our study. However, the supportive effects of anti-CTLA-4 or anti-PD-L1 therapies on treatment with cisplatin or a synthetic fusion protein vaccine, inducing an immune response against the HPV16 E7 oncoprotein, respectively, have been previously shown in TC-1-induced tumors [45,46]. Similarly to the TC-1/dCD80-1-induced tumors, anti-CTLA-4 administered as a single therapy reduced the progression of another tumor type, i.e., mouse bladder cancer induced by the MB49 cell line with low CD80 expression [6,47].

Unlike in our study, simultaneous blockade of CTLA-4/CD80 and PD-1/PD-L1 axes has been more efficient than a single therapy in several studies [48,49,50]. Paradoxically, anti-PD-L1 administered as a single therapy may enhance CTLA-4/CD80-mediated immunosuppression in some patients due to the disruption of the tumor-suppressive CD80 and PD-L1 in cis interaction [9]. However, the interaction of mouse CTLA-4 with CD80 has been reported to outcompete the tumor-suppressive CD80 and PD-L1 in cis interaction [51]. This corresponds to our previous observation showing that deactivation of PD-L1 in the TC-1 cell line markedly reduced the tumorigenicity of these cells, which implies the pro-tumorigenic role of the PD-L1 molecule, rather than CD80/PD-L1-mediated tumor suppression [52]. We, therefore, assume that PD-L1 on tumor cells did not effectively control the CTLA-4/CD80 axis in TC-1- and TC-1/dCD80-1-induced tumors. 

In our study, the depletion of CD8^+^ cells abrogated the effect of CTLA-4 blockade, whereas the depletion of CD4^+^ cells synergized with the therapy in TC-1/dCD80-1-induced tumors. This result corresponds to published data showing that the efficacy of anti-CTLA-4 treatment has been dependent on activated tumor-infiltrating CD8^+^ T cells [53]. Moreover, distinct levels of PD-1, a marker of T cell activation, on CD8^+^ T cells [54,55], might also support the enhanced efficacy of CTLA-4 blockade in TC-1/dCD80-1- compared with that in TC-1-induced tumors. Furthermore, we did not observe a significant impact of the depletion of NK1.1^+^ cells or macrophages on the efficacy of CTLA-4 blockade although this treatment has been shown to induce the activation and degranulation of tumor-infiltrating NK cells [56].

CTLA-4 or PD-L1 blockade downregulated the frequency of immune cells in TC-1-induced tumors, while anti-CTLA-4 treatment upregulated the frequency of most lymphoid cells in TC-1/dCD80-1-induced tumors. Enhanced infiltration of immune cells into tumors treated with immune checkpoint inhibitors has been previously reported [57]. As the efficacy of immune checkpoint blockade is dependent on tumor-infiltrating immune cells and tumor-specific T cell responses [58], increased infiltration of TC-1/dCD80-1-induced tumors by immune cells might contribute to the sensitivity of these tumors to anti-CTLA-4 therapy. 

The CD4^+^ T cell subsets regulate tumor growth differently [59]. Th1 cells in tumors protect the host against tumor growth, whereas Th17 and Treg cells are usually associated with progression of the disease, and Th2 cells do not correlate with clinical outcomes in many cases [59,60,61,62,63]. The anti-CTLA-4 treatment upregulated the frequency of Th1, Th2, Th17, and Treg cells in TC-1/dCD80-1-induced tumors. This effect was not elicited in TC-1-induced tumors. The highly increased Th1 cells probably contributed to the anti-tumor effect of CTLA-4 blockade in TC-1/dCD80-1-induced tumors. Similarly, CTLA-4 blockade has been previously reported to enrich Th1 and Th2 subsets in mouse as well as human tumors and enhance IFN-γ production by T cells [64,65,66].

Treg cells comprised the most abundant CD4^+^ T cell subset in both TC-1- and TC-1/dCD80-induced tumors. Treg subpopulations 1 and 2, automatically generated by the FlowSOM software, markedly expressed CTLA-4, GITR, and ICOS molecules. High expression of CTLA-4, as well as co-stimulatory receptors GITR and ICOS, has been observed in Treg cells infiltrating mouse and human tumors [26]. CTLA-4 has been shown to be indispensable in Treg-mediated immunosuppression, as CTLA-4^−^ Treg cells were unable to maintain self-tolerance and immune homeostasis, and Treg-specific CTLA-4 deactivation promoted anti-tumor immunity [19]. Moreover, GITR and ICOS molecules maintain Treg homeostasis, survival, and immunosuppressive functions [67,68,69]. Thus, we suppose that the immunosuppressive potential of subpopulations 1 and 2 was high, whereas the low CTLA-4 expression in subpopulations 3 and 4 implied limited immunosuppressive potential. Furthermore, subpopulation 4 of Treg cells was characterized by a low expression of ICOS and GITR and slightly higher Nrp-1 expression compared with that in the remaining Treg subpopulations. Similarly, Nrp-1 did not cluster with other markers in a study of the phenotypic diversity of Treg cells isolated from skin [70]. Nrp-1^+^ Treg cells have a strong potential to infiltrate tumors in a vascular endothelial growth factor (VEGF)-dependent manner and inhibit the anti-tumor immune response [25]. As previously noted, VEGF occurs in TC-1-induced tumors [71]. However, the ICOS^−^ Treg subset has been defined as “death prone” [67,68]. Therefore, we presume that the immunosuppressive potential of subpopulation 4 was markedly limited. Our analysis of Treg subpopulations suggests that the decreased immunosuppressive potential of Treg cells in TC-1/dCD80-1-induced tumors after CTLA-4 blockade might also contribute to the anti-tumor effect of anti-CTLA-4 therapy in these tumors.

## 4. Materials and Methods

### 4.1. Mice

Animal experiments were performed with female 6 to 8-week-old C57BL/6N mice (Charles River, Sulzfeld, Germany) that were maintained under specific pathogen-free conditions at the animal facility of the Czech Center of Phenogenomics (BIOCEV, Vestec, Czech Republic). The guidelines for the proper treatment of laboratory animals were observed.

### 4.2. Cells and CD80 Deactivation

The TC-1 cell line (Cellosaurus ID: CVCL_4699; provided by T.-C. Wu, John Hopkins University, Baltimore, MD, USA) was derived from C57BL/6 mouse primary lung cells by transformation with HPV16 *E6*/*E7* and human *H-ras* oncogenes [72]. 

The B7-1 Double Nickase Plasmid (m) kit (sc-419570-NIC; Santa Cruz Biotechnology, Dallas, TX, USA) was used to produce TC-1/dCD80 cell clones with deactivated CD80. The transfected cells were selected for 4 days with 6 μg/mL puromycin, which was added to the culture media 2 days after transfection. Next, cells were stained with anti-CD80-FITC (fluorescein isothiocyanate) antibody (clone 16-10A1; BD Pharmingen, San Diego, CA, USA) and single-cell clones with deactivated CD80 were selected by cell sorting into a 96-well plate using a flow cytometer FACSAria Fusion (BD Biosciences, Franklin Lakes, NJ, USA). Deactivation of the *CD80* gene in the TC-1/dCD80-1 clone was verified by sequencing of the target site.

Cell lines were cultured in high-glucose Dulbecco’s modified Eagle’s medium (DMEM; Sigma-Aldrich, Merck KGaA, Darmstadt, Germany) containing 10% fetal calf serum (FCS; Biosera, Nuaille, France) and supplemented with 100 μg/mL streptomycin and 100 IU/mL penicillin (Biosera).

### 4.3. Animal Experiments

Tumor cells were suspended in 150 µL PBS and subcutaneously (s.c.) administered into the back of C57BL/6N mice (five per group) under anesthesia with xylazine (16 mg/kg; Bioveta, Ivanovice na Hane, Czech Republic) and ketamine (100 mg/kg; Bioveta). Tumors were measured three times a week with calipers, and tumor volume was calculated using the formula (π/6) (a × b × c), where a, b, and c are tumor dimensions.

Animals received intraperitoneal (i.p.) treatment with anti-CTLA-4 (150 µg, clone 9D9) monoclonal antibody (Bio X Cell, West Lebanon, NH, USA) on days 3, 6, and 10 after tumor cell inoculation, or anti-PD-L1 antibody (200 µg, clone 10F.9G2) on days 17, 20, and 24 after tumor cell inoculation. For flow cytometry analysis of the tumor microenvironment, mice were treated with anti-PD-L1 on days 10, 13, and 17 after tumor cell administration.

Depletion of CD4^+^, CD8^+^, and NK1.1^+^ cells in vivo was achieved by monoclonal antibodies (Bio X Cell) diluted in 200 μL PBS and injected i.p. (100 µg of anti-CD4 (clone GK1.5) with 100 µg of anti-CD8 (clone 2.43), and 100 µg of anti-NK1.1 (clone PK136)) twice a week. Macrophages were depleted by i.p. injection of 1 mg of carrageenan IV (Sigma-Aldrich, Merck KGaA) dissolved in 200 µL PBS twice a week. The initial dose of antibodies and carrageenan was administered 3 days after tumor cell inoculation. Depletion efficacy was verified by the examination of splenocytes.

### 4.4. Stimulation of Cells Isolated from Tumors

Single-cell suspensions were prepared from tumors as previously described [52] and stimulated prior to staining with panels of antibodies for flow cytometry analysis of the T cells. In total, 2.5 × 10^6^ cells were cultivated for 3 hours in 2 mL of Roswell Park Memorial Institute (RPMI) 1640 medium (Sigma-Aldrich, Merck KGaA) supplemented with 10% FCS (Biosera), 100 IU/mL penicillin, 100 μg/mL streptomycin (Biosera), and 50 µM 2-mercaptoethanol and containing 81 nM phorbol 12-myristate 13-acetate, 1.34 µM ionomycin, 2 µM monensin, and 10.6 µM brefeldin A (Abcam, Cambridge, UK). 

### 4.5. Flow Cytometry

Cells obtained from tumors were incubated with Fixable Viability Dye eFluor 455UV (eBioscience) to stain dead cells. Then, the cells were treated with anti-mouse CD16/32 (Fc block, clone 93; BioLegend) and subsequently with antibodies binding surface markers (Table 1). The washed cells were fixed and permeabilized with the Fixation/Permeabilization working solution (eBioscience, Thermo Fisher Scientific, Waltham, MA, USA). Furthermore, a working solution of the Permeabilization Buffer (eBioscience) was used to stain intracellular and nuclear markers with respective antibodies. Measurement of the stained samples was performed on LSRFortessa (BD Biosciences, San Diego, CA, USA) and CytoFLEX LX (Beckman Coulter, Indianapolis, IN, USA) flow cytometers. FlowJo™ software version 10.7 (BD Biosciences), FlowSOM version 2.6 [73], and R version 4.0.2 were used for data analysis. Gating strategies are depicted in Figures S3 and S4. The values of the parameters were as follows for the calculation of t-SNE: iterations—1000, perplexity—30, learning rate (Eta)—478, gradient algorithm—Barnes–Hut; and, for FlowSOM, number of meta clusters: 4; set seed: 3.

### 4.6. Statistical Analysis

Flow cytometry analysis and animal experiments were evaluated by two-way analysis of variance and Bonferroni’s post-test using Prism software, version 7 (GraphPad Software, San Diego, CA, USA). A difference of results was considered significant if *p* < 0.05.

## 5. Conclusions

CD80 expression on TC-1 tumor cells affected the tumor microenvironment and sensitivity to immunotherapy. CD80 deactivation in these cells was associated with a “hotter” microenvironment, decreased tumor growth, and enhanced sensitivity to CTLA-4 blockade. The impact of CD80 expression on tumor cells on the efficacy of CTLA-4 blockade has not been sufficiently investigated yet. Our study implies that CD80 expression on tumor cells should be evaluated further as a possible predictive marker that may assist clinicians in the selection of cancer patients who may be suitable for CTLA-4 blockade cancer therapy. Finally, the development of the tumor-cell-targeted CD80 blockade should be assessed as a novel immunotherapeutic approach.

## Figures and Tables

**Table 1:** Antibodies for flow cytometry.

Antigen	Conjugate	Clone	Source	Staining	Panels			
CD11b	BV421	M1/70	BioLegend	Surface		1		
CD11c	APC-Cy7	N418	BioLegend	Surface		1		
CD25	APC	PC61.5	eBiosciences	Surface	1		1	1
CD3	APC-Cy7	145-2C11	BioLegend	Surface	1		1	1
CD317	APC	927	BioLegend	Surface		1		
CD4	BV510^1^/PerCP-Cy5.5^2^	RM4-5	BioLegend	Surface	1		2	2
CD45	Alexa Fluor 700	30-F11	BioLegend	Surface	1	1	1	1
CD73	BV605	TY/11.8	BioLegend	Surface				1
CD8	FITC	53-6.7	BD Biosciences	Surface	1		1	
CTLA-4	PE/Dazzle594	UC10-4B9	BioLegend	Surface			1	1
F4/80	BV510	BM8	BioLegend	Surface		1		
Foxp3	PE	FJK-16s	eBiosciences	Nuclear	1		1	1
GITR	FITC	DTA-1	BioLegend	Surface				1
Granzyme B	PE/Cy7	NGZB	BioLegend	Intracellular			1	1
ICOS	BV650	7E.17G9	BioLegend	Surface				1
IFN-γ	BV421	XMG1.2	BioLegend	Intracellular			1	
IL-17A	BV650	TC11-18H10.1	BioLegend	Intracellular			1	
IL-4	BV786	11B11	BD Biosciences	Intracellular			1	
Lag3	BV785	C9B7W	BioLegend	Surface				1
Ly6C	BV786	HK1.4	BioLegend	Surface		1		
Ly6G	FITC	1A8	BioLegend	Surface		1		
MHC-II	PE-Cy7	114.15.2	BioLegend	Surface		1		
NK1.1	BV650	PK136	BioLegend	Surface	1			
Nrp-1	BV 421	3E12	BioLegend	Surface				1
PD-1	PE-Cy7^1^/PE^2^	29F.1A12	BioLegend	Surface	1	2		
PD-L1	PE-DAZZLE 594 ^1^/BV650 ^2^	10F.9G2	BioLegend	Surface	1	2		
TCR γ/δ	BV605	GL3	BioLegend	Surface	1		1	

^1,2^—antibody present in a panel.

## Data Availability

Data is contained within the article or Supplementary Material.

## Supplementary Materials

The following are available online at https://www.mdpi.com/article/10.3390/cancers13081935/s1, Figure S1: Surface expression of PD-1 by leucocytes infiltrating TC-1- and TC-1/dCD80-induced tumors. Figure S2: Expression of Treg markers on the FlowSOM-identified subpopulations 1–4. Figure S3: Gating strategy for lymphoid subpopulations. Figure S4: Gating strategy for myeloid subpopulations.
