# Supplementary figures and images for: CD80 Expression on Tumor Cells Alters Tumor Microenvironment and Efficacy of Cancer Immunotherapy by CTLA-4 Blockade
Source: Cancers (Basel). 2021 Apr 16;13(8):1935. doi: 10.3390/cancers13081935 (PMC8072777; doi:10.3390/cancers13081935)

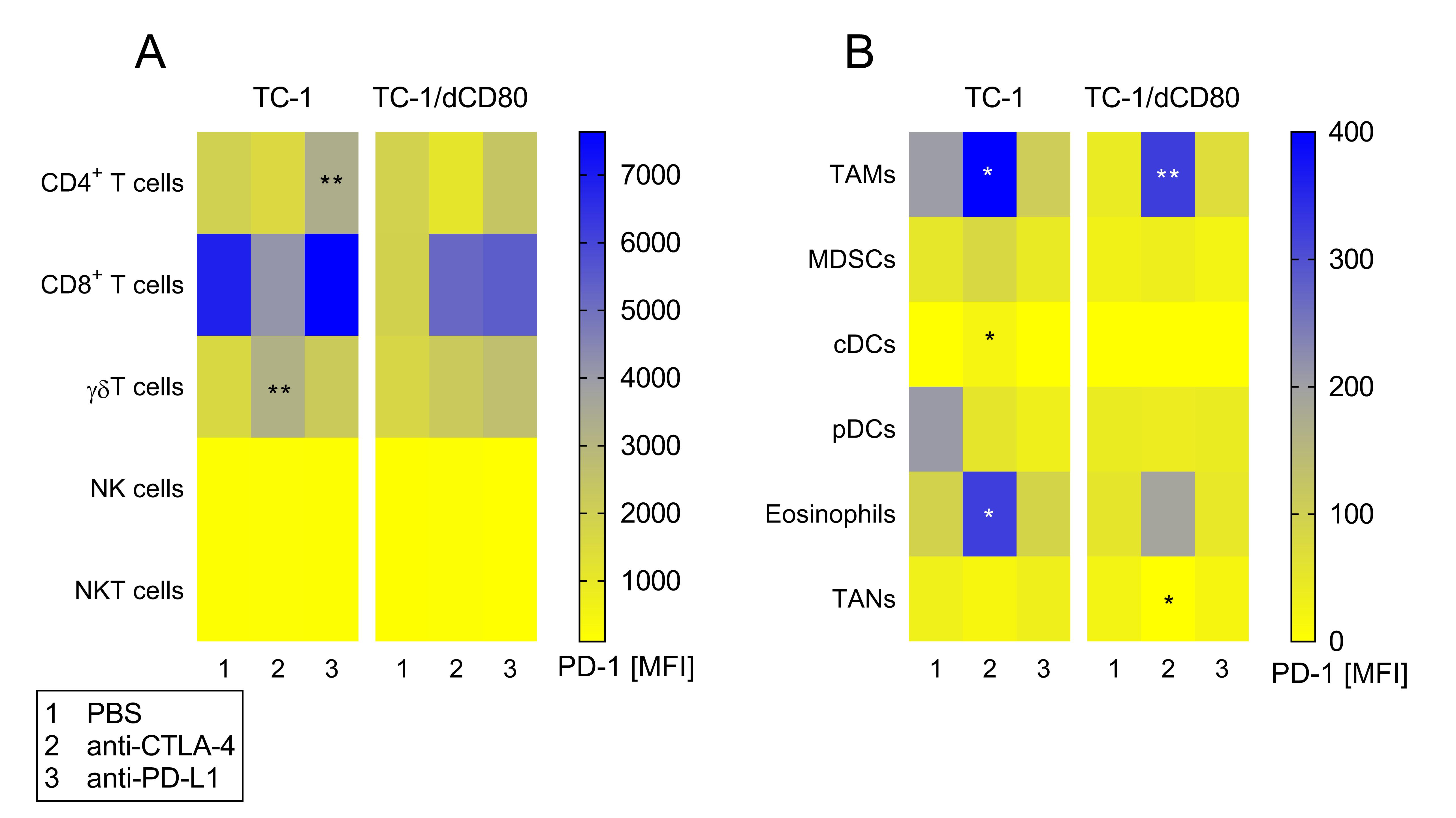

Supplement: Supplementary file 1 [file cancers-13-01935-s001.zip › Figure S1.jpg]

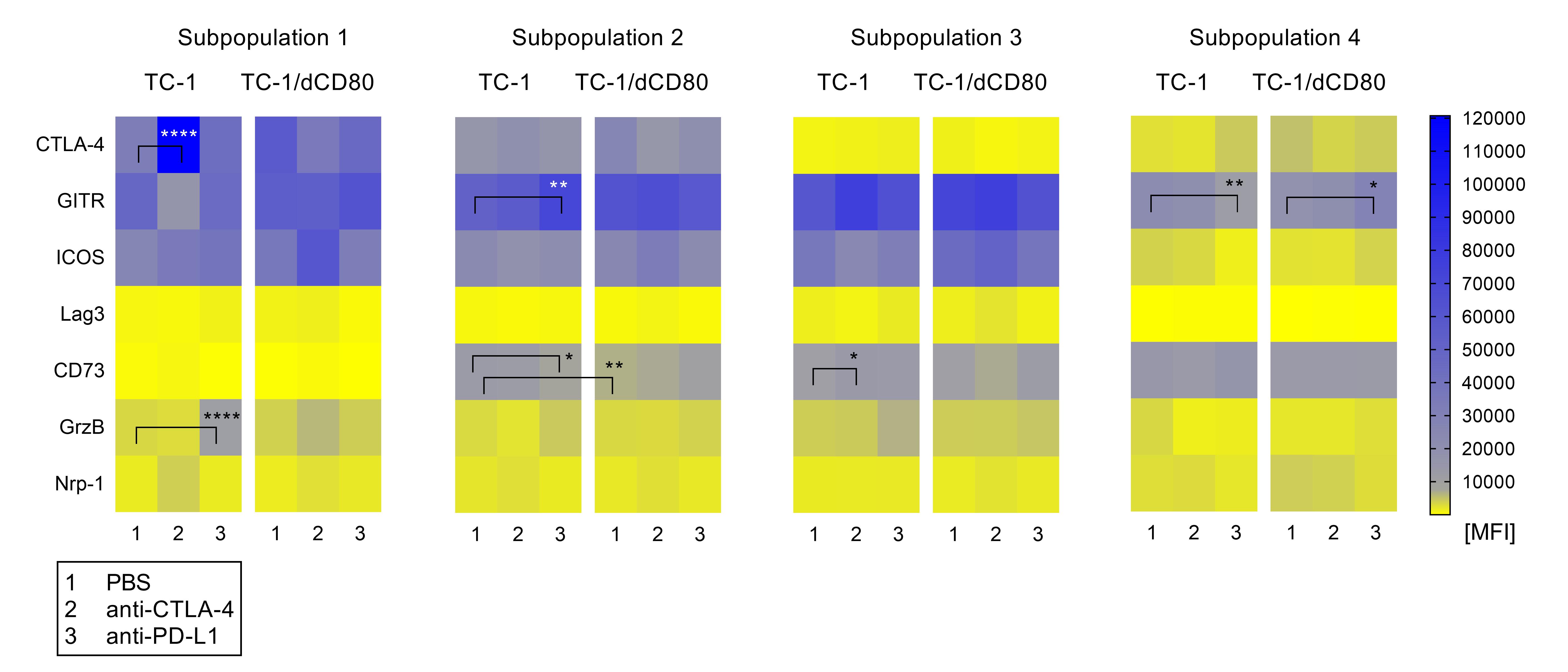

Supplement: Supplementary file 1 [file cancers-13-01935-s001.zip › Figure S2.jpg]

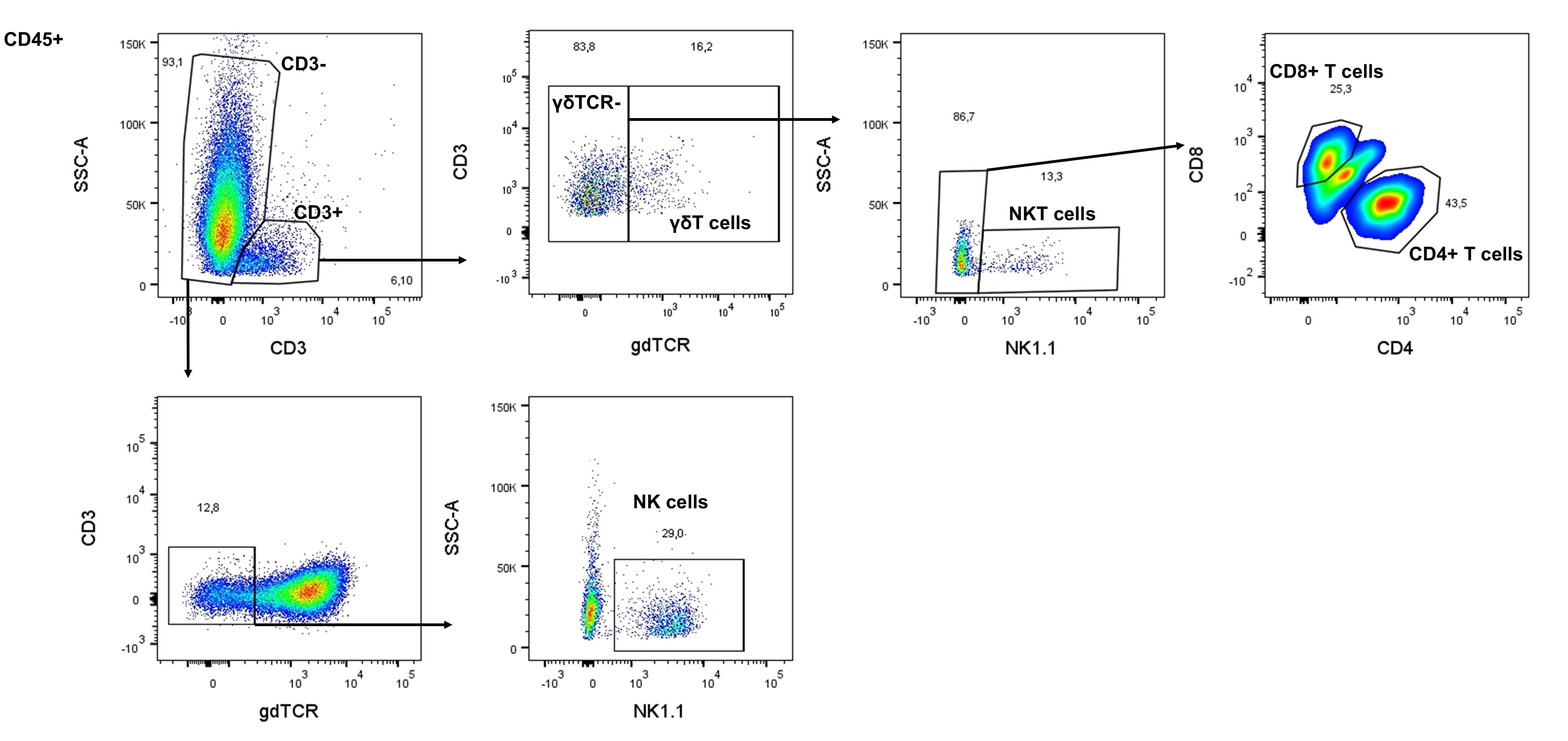

Supplement: Supplementary file 1 [file cancers-13-01935-s001.zip › Figure S3.jpg]

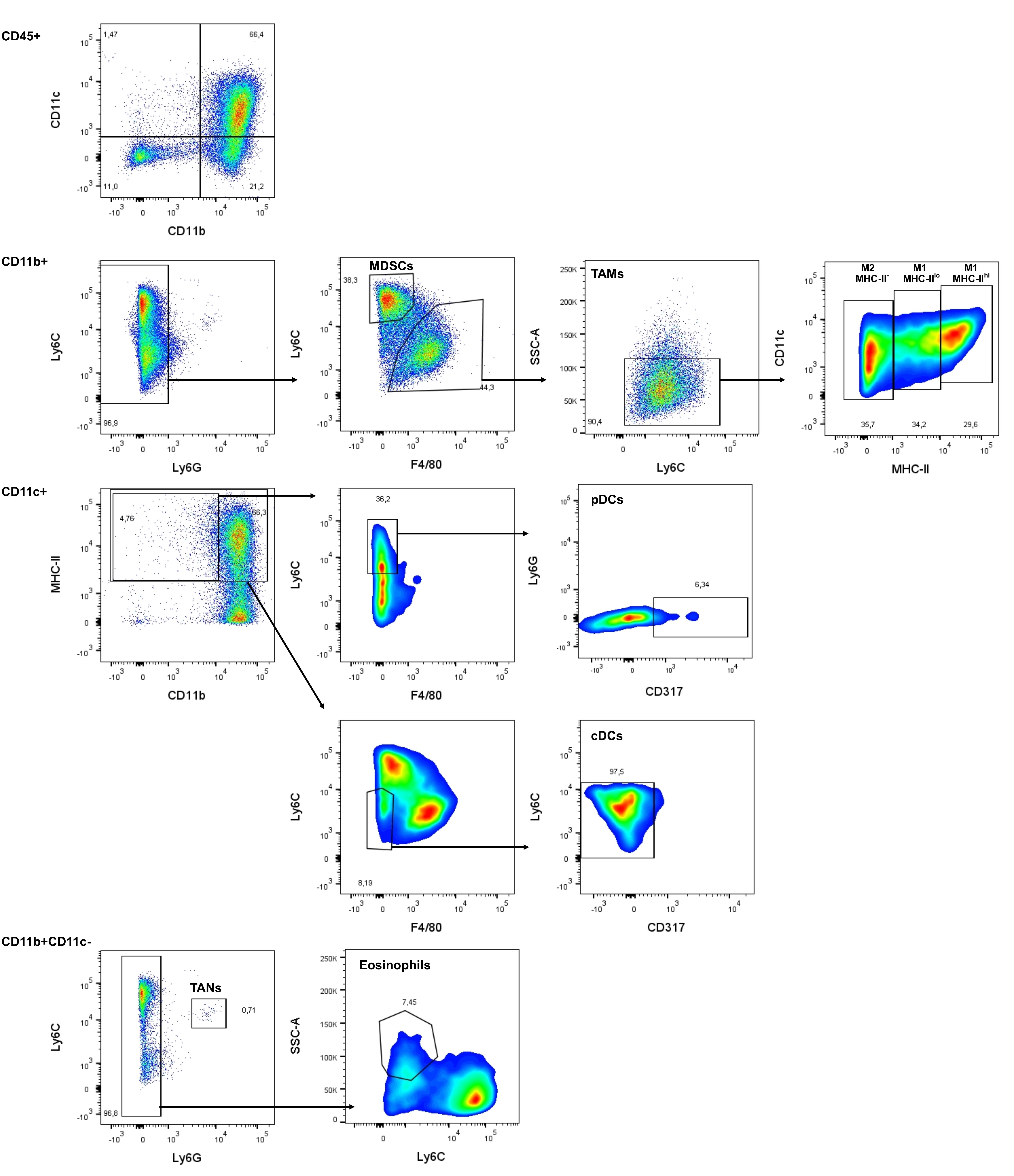

Supplement: Supplementary file 1 [file cancers-13-01935-s001.zip › Figure S4.jpg]
